# Associations between social determinants and the presence of chronic diseases: data from the osteoarthritis Initiative

**DOI:** 10.1186/s12889-020-09451-5

**Published:** 2020-08-31

**Authors:** Vishal Vennu, Tariq A. Abdulrahman, Aqeel M. Alenazi, Saad M. Bindawas

**Affiliations:** 1 Department of Rehabilitation Sciences, College of Applied Medical Sciences, King Saud University, Riyadh, 10219 Saudi Arabia; 2 Department of Rehabilitation Sciences and Physical Therapy, Prince Sattam Bin Abdulaziz University, Alkharj, Saudi Arabia

**Keywords:** Social determinant, chronic diseases, arthritis, Asthma, Cancer, COPD, Diabetes, Heart attack, Caucasian, African American

## Abstract

**Background:**

Some studies investigated the relationship between musculoskeletal conditions and chronic diseases. However, no study examined the association between social determinants and chronic diseases among people at high risk for knee osteoarthritis. Thus, the current study was aimed to address this gap.

**Methods:**

A secondary data analysis was conducted on a total of 3280 men and women aged 45 to 79 who were recruited in the Osteoarthritis Initiative.

**Results:**

Multivariable logistic regression analyses show that age ≥ 65 years was associated with 1.98, 1.96, and 1.46 times odds of the presence of diabetes, heart attack, and multi-morbidity, respectively than age ≤ 64 years. Men were associated with 1.39, 1.41, 1.76, and 2.24 times odds of the presence of arthritis, cancer, diabetes, and heart attack, respectively than women. African American/Asian/ non-Caucasian was associated with 2.71, 2.56, and 1.93 times odds of the presence of arthritis, diabetes, and heart attack, respectively than Caucasian. Primary school/less education was associated with twice or more times the odds of arthritis and chronic obstructive pulmonary disease (COPD) than ≥high school education. Unemployment was associated with 1.41-, 1.73-, 1.58-, and 1.70-time odds of the presence of arthritis, cancer, COPD, and heart attack, respectively, then employed. Unmarried/widowed/separated was associated with 1.41, 1.75, 2.77, 2.76, 1.86, and 3.34 times odds of the presence of arthritis, asthma, cancer, COPD, diabetes, and heart attack, respectively than married. Annual income < 50,000 was associated with 1.33-, 1.44-, and 1.38-time odds of the presence of arthritis, diabetes, and multi-morbidity, respectively, then annual income ≥50,000. Overweight/obese was associated with 2.28 times the odds of the presence of diabetes than healthy weight. Current/former smoker was associated with 1.57, 2.47, 2.53, 1.63, and 1.24 times odds of the presence of arthritis, cancer, COPD, heart attack, and multi-morbidity, respectively than a nonsmoker. Consuming alcohol was associated with 1.32-, 1.65-, 1.50-, and 1.24-time odds of the presence of arthritis, COPD, diabetes, and multi-morbidity, respectively, then nonalcoholic.

**Conclusions:**

Social determinants are associated with the presence of chronic diseases. Some of the social determinants are modifiable or treatable. Thus, these findings can inform public health strategies in the United States.

## Background

The World Health Organization defines social determinants as “the conditions in which people are born, grow, work, live, and age and the set of forces and systems shaping the conditions of daily life” [1]. Some social determinants, such as income, education, occupation, racial discrimination, smoking, and alcohol consumption, affect individuals, groups, and communities positively or negatively [2]. These modifiable social determinants can have a causal role in promoting illness conditions, such as chronic diseases and disability, that may continue to increase if not adequately addressed [3, 4].

Chronic diseases are broadly described as “conditions that last two years or more and require ongoing medical attention or limit activities of daily living or both” [5]. Some chronic diseases—like heart disease, cancer, and diabetes —are the leading cause of long-term disability, reduced quality of life (QoL), and death (7 out of 10) in the United States (US) [5, 6]. One in two adults have a chronic disease, and one in four adults has multiple chronic conditions [5]. These play a crucial role in the 2.7 trillion dollars spent on national healthcare annually [5]. Currently, the top ten health problems in the US are due to an overall increase in chronic diseases, especially heart disease, cancer, and diabetes [7], which could be associated with social determinists.

Social determinants can initiate the onset of pathology and serve as a direct problem for some chronic diseases. For example, smoking is associated with more than 21 chronic conditions [8]. Other social determinants such as income, education, occupational characteristics, and racial inequality have direct effects on unhealthy and healthy lifestyles and connections to chronic diseases [3, 9, 10]. Several earlier studies among the various population have revealed that the association between social determinants and chronic diseases [11], especially cardiovascular diseases [12–14] and diabetes [15]. Some of these studies have been criticized due to their nature and inadequate control of confounding factors. A study reviewed social determinants’ contributions to the historical declines in cardiovascular mortality rate [16]. That study has concluded that understanding patterns, trends of social inequalities in cardiovascular disease, and risk factors are required across the life course in different settings.

Some researchers have found that social determinants are potent determinants of health outcomes [17–20]. However, no study has determined the relationship between social determinants and chronic diseases in people at high risk for knee osteoarthritis [21, 22]. Therefore, the current study was aimed to address this gap by examining the association between social determinants and the presence of chronic diseases among this population.

## Methods

### Data

A secondary analysis was conducted utilizing data from the Osteoarthritis Initiative (OAI; released version 0.2.2). The OAI is a public and privately funded large multi-center ten-year observational cohort study. The OAI is available for open access freely at the United States’ National Institute of Health data repository [23]. The OAI enrolled men and women aged 45–79 years (regardless of race/ethnicity) with or at high risk for knee osteoarthritis. Data were collected at four clinical sites in the US (Baltimore, MD; Pittsburgh, PA; Pawtucket, RI; and Columbus, OH) between February 2004 and May 2006. The OAI’s primary exclusion criteria were inflammatory arthritis, contraindication to 3 T MRI., and bilateral end-stage knee osteoarthritis.

The Institutional Review Board of a coordinating center, University of California, San Francisco, approved the OAI’s protocol. All participants gave informed written consent before joining.

### Study design and participants

In this study, 3280 men and women aged 45—79 years with a high risk for knee osteoarthritis were included after excluding missing data (*n* = 4), the progression, and control sub cohorts. High risk for knee osteoarthritis was defined as no symptomatic tibiofemoral knee osteoarthritis at baseline but had an elevated risk of developing knee osteoarthritis symptoms during the study. The progression sub-cohort was defined as participants with symptomatic tibiofemoral knee osteoarthritis at baseline. The control sub cohort defied as participants with no pain, aching, or stiffness in either knee in the past year, along with no radiographic findings of osteoarthritis and no eligibility risk factors. Participants’ socio-demographics, body mass index (BMI), smoking status, and alcohol intake were collected. The data cleaning was performed according to the general guidelines for conducting secondary analyses of existing data [24]. The OAI was a 10-year observational cohort study. Using OAI data, several studies have been published to date. Therefore, we believe that this data can help explain the association even if it was collected 14-years back due to the participants following up to 10-year.

### Measures

Based on self-report, social determinants were assessed and dichotomized as follows: age (≥ 65 vs. < 65 years), sex (men vs. women), race (African Americans, Asians, or other non-Caucasians vs. Caucasians), education (≤ primary school or less vs. ≥ high school or more), employment status (unemployed vs. employed), marital status (unmarried, widowed, or separated vs. married), and household composition (living alone vs. others), personal annual income (< 50,000 vs. ≥ 50,000 US dollars); BMI (obese vs. normal weight), smoking (current or former vs. never), and alcohol consumption (yes vs. no). The annual income was dichotomized into two levels based on the US Census Bureau’s statistics about the yearly median personal income [25]. BMI was dichotomized into two levels: normal weight (BMI = 18.5–24.9 kg/m2) and overweight/obese (BMI ≥ 25 kg/m2) [26]. The Charlson comorbidity index (CCI) was used to assess the comorbidity conditions of chronic diseases, such as arthritis, asthma, cancer, chronic obstructive pulmonary diseases (COPD), diabetes, and heart attack. Also, CCI was used to assess multi-morbidity defined as two or more chronic diseases. The CCI has been widely used to distinguish comorbidity conditions [27].

### Statistical analyses

Descriptive statistics for all participants were calculated in frequencies and percentages. Multivariable logistic regression analyses were utilized to examine the associations between social determinants, each chronic disease, and multi-morbidity. Social determinants were the independent variables, whereas chronic diseases and multi-morbidity were the dependent variables. Each model was adjusted for social determinants for each chronic condition and multi-morbidity. All analyses were conducted utilizing Statistical Analysis Software, version 9.2 (SAS corporation Inc., Cary, NC, US) for Windows.

## Results

**Fig. 1:**
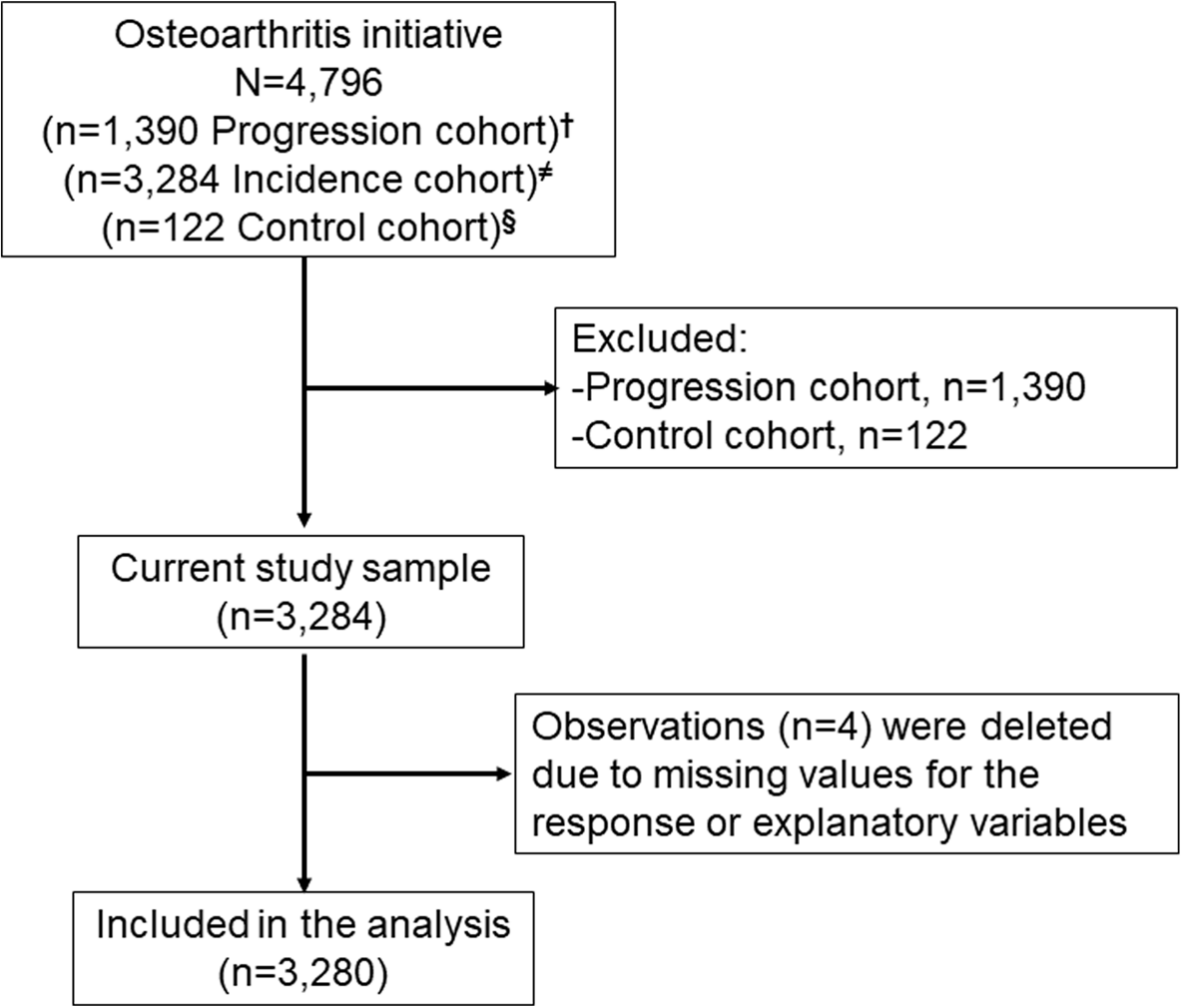
Participant flow. ^**†**^Participants with symptomatic tibiofemoral knee osteoarthritis at baseline. ^**≠**^Participants with no symptomatic tibiofemoral osteoarthritis in either knee at baseline, but had an elevated risk of developing symptoms of knee osteoarthritis during the study. ^**§**^Participants with no pain, aching, or stiffness in either knee in the past year, along with no radiographic findings of osteoarthritis and no eligibility risk factors

**Fig. 2:**
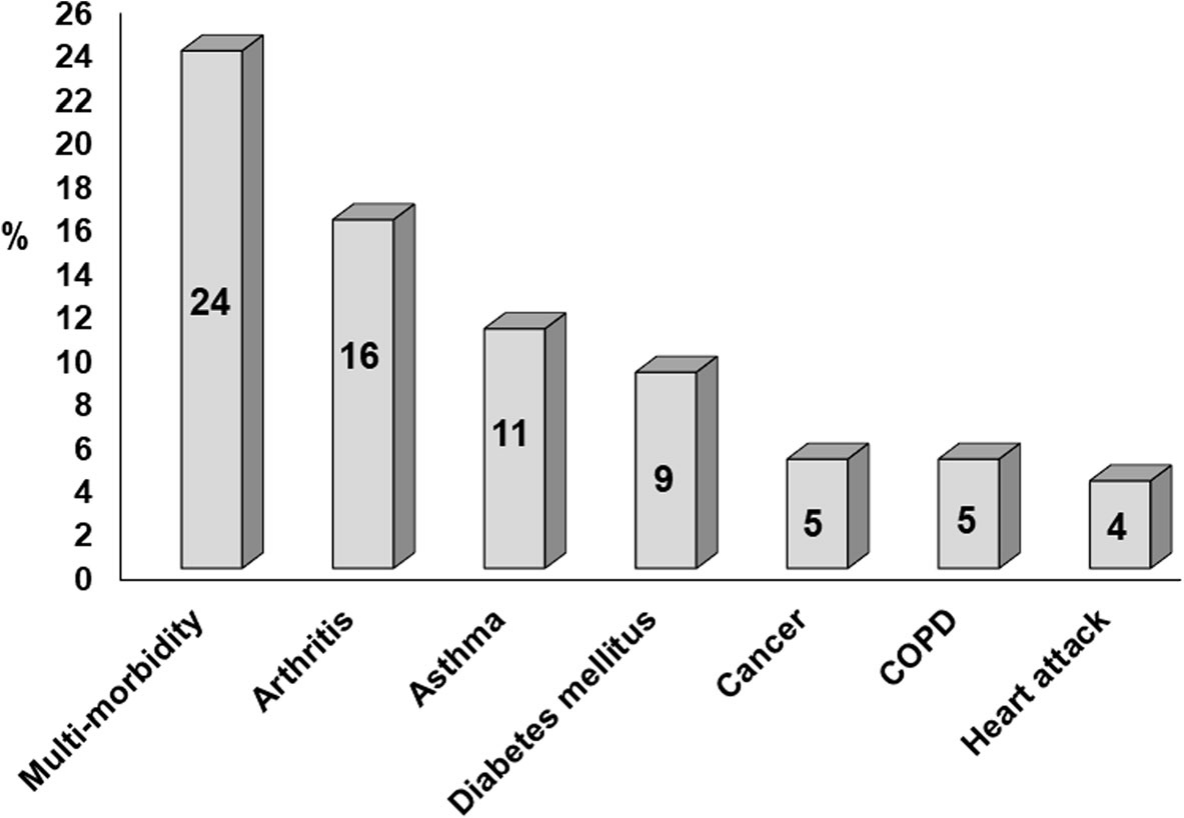
The frequency of each chronic disease and multi-morbidity. COPD = chronic obstructive pulmonary disease

Figure 1 shows the flow of the study participants. Only data from four participants were not included in analyses due to missing values after excluding the progression (*n* = 1390), and control (*n* = 122) sub-cohorts. Descriptive statistics for all study participants are shown in Table 1. The frequency of each chronic disease is illustrated in Fig. 2.

**Table 1:** Baseline descriptive statistics of all study participants, *n* = 3280

Variables	Frequency	Percentage
Age (years)		
< 65	2020	62
≥ 65	1260	38
Sex		
Man	1348	41
Woman	1932	59
Race		
Caucasian	2703	82
African American/Asian/Other	577	18
Educational status		
≥ High school	3183	97
≤ Primary school	97	3
Employment status		
Employed	2004	61
Unemployed	1276	39
Marital status		
Married	2200	67
Unmarried/widowed/divorced	1080	33
Household composition		
Live with others (spouse, children, or relatives)	2560	78
Live alone	720	22
The personal income per annum in US dollars		
≥ 50,000	2115	65
> 50,000	1165	35
Body mass index		
Normal weight (18.5–25 kg/m2)	880	27
Overweight/obese (≥25 kg/m2)	2391	73
Smoking history		
Never smoked	1581	48
Current or former smoker	1699	52
Alcohol consumption		
No	1512	46
Yes	1768	54

The associations between social determinants, each chronic disease, and multi-morbidity are presented in Table 2. Adults aged ≥65 years were more likely to have diabetes, a heart attack, and multi-morbidity than were their younger counterparts. Men more likely to have arthritis, cancer, diabetes, and heart attack compared to a woman. African Americans, Asians, other non-Caucasian were more likely to have arthritis, diabetes, or a heart attack than were Caucasians. Those who were unmarried, widowed, or separated were more likely to have arthritis, Asthma, cancer, COPD, diabetes, or a heart attack than were those who were married. Also, those with a primary school or less education were more likely to have arthritis or COPD than their more-educated counterparts. Unemployed participants were more likely to have arthritis, cancer, COPD, or a heart attack than those employed. Those with a <  50,000 US dollars’ annual income were more likely to have arthritis, diabetes, multi-morbidity than were those who earned more than equal to 50,000 US dollars.

**Table 2:** Associations between social determinants and the presence of each chronic disease and multi-morbidity among adults, *n* = 3280

Social determinant	Chronic disease						
	Arthritis	Asthma	Cancer	COPD	Diabetes	Heart attack	Multi-morbidity^a^
	OR (95% CI)	OR (95% CI)	OR (95% CI)	OR (95% CI)	OR (95% CI)	OR (95% CI)	
Age (≥ 65 vs. < 65 years)	0.92 (0.73–1.15)	0.61 (0.47–0.80)	1.24 (0.86–1.80)	1.12 (0.76–1.64)	1.98* (1.48–2.65)	1.96** (1.30–2.95)	1.46** (1.17–1.83)
Sex (Men vs. Women)	1.39** (1.14–1.70)	0.72 (0.57–0.92)	1.41*** (1.02–1.96)	1.37 (0.97–1.94)	1.76* (1.36–2.29)	2.24* (1.56–3.23)	1.06 (0.89–1.27)
African American/Asian/Other vs. Caucasian	2.71* (2.23–3.48)	1.00 (0.75–1.34)	1.22 (0.81–1.83)	1.40 (0.94–2.10)	2.56* (1.92–3.42)	1.93** (1.28–2.91)	0.58 (0.47–0.71)
≤ Primary school vs. ≥ high school	2.00** (1.27–3.13)	1.70 (0.98–2.95)	0.81 (0.34–1.94)	2.15*** (1.12–4.12)	1.14 (0.64–2.02)	0.96 (0.43–2.12)	0.74 (0.61–0.92)
Unemployed vs. employed	1.26*** (1.01–1.57)	1.23 (0.95–1.59)	1.73** (1.21–2.48)	1.58*** (1.10–2.30)	1.13 (0.86–1.50)	1.70** (1.15–2.53)	0.62 (0.52–0.75)
Unmarried/widow/divorced vs. married	1.41*** (1.06–1.87)	1.75** (1.26–2.42)	2.77** (1.79–4.29)	2.76* (1.76–4.32)	1.86** (1.31–2.64)	3.34* (2.13–5.23)	1.11 (0.80–1.45)
Live alone vs. live with others	0.77 (0.57–1.05)	0.67 (0.47–0.96)	0.52 (0.33–0.84)	0.51 (0.31–0.82)	0.57 (0.39–0.83)	0.28 (0.67–0.47)	1.08 (0.80–1.45)
Annual Income in USD (< 50,000 vs. ≥ 50,000)	1.33*** (1.07–1.65)	1.12 (0.87–1.45)	0.94 (0.66–1.35)	1.05 (0.72–1.52)	1.44** (1.09–1.89)	1.19 (0.81–1.74)	1.38* (1.09–1.75)
Overweight/obese vs. normal weight	1.31 (0.96–1.79)	1.44 (0.98–2.11)	0.91 (0.55–1.49)	1.07 (0.65–1.76)	2.28** (1.43–3.63)	1.33 (0.77–2.30)	0.67 (0.55–0.80)
Current/former smoker vs. never	1.57** (1.20–2.05)	1.16 (0.85–1.59)	2.47** (1.52–4.00)	2.53** (1.58–4.05)	1.05 (0.75–1.47)	1.63*** (1.02–2.58)	1.24** (1.02–1.51)
Alcohol consumption (yes vs. no)	1.32*** (1.02–1.72)	1.16 (0.84–1.58)	1.45 (0.93–2.27)	1.65*** (1.07–2.54)	1.50*** (1.08–2.10)	1.54 (0.98–2.41)	1.24*** (1.01–1.52)

Each model for social determinant factor was adjusted for other social determinants

*COPD* Chronic obstructive pulmonary disease, *OR* Odds ratio, *CI* Conference interval

*p < .001; **p = .001; ***p < .05

^**a**^Multi-morbidity was defined as two or more chronic diseases, such as arthritis, asthma, cancer, COPD, diabetes, and heart attack

Those who were overweight/obese were more likely to have diabetes than were those who were a healthy weight. Participants who smoked or had smoked were more likely to have arthritis, cancer, COPD, a heart attack, and multi-morbidity than were those who never smoked. Lastly, those who consumed alcohol were more likely to have arthritis, COPD, diabetes, and multi-morbidity than were those who did not drink alcohol (Table 2).

## Discussion

The current study examined the association between social determinants and the presence of chronic diseases among people at high risk for knee osteoarthritis. The results showed that social determinants, such as being aged ≥65 years, men, African Americans, Asians, or other non-Caucasians; unmarried, widowed, or separated; less educated, being unemployed, having a <  50,000 US dollar annual income, being overweight or obese, being a current or former smoker, and consuming alcohol were associated with the presence of several chronic diseases in this population. No other study had evaluated the significance of these selected social determinants on the association of chronic diseases among this community [21].

Consistent with the present findings, it has been reported that aging is disproportionately associated with chronic diseases, contributing to reduced QoL, increased disability, and increased long-term healthcare costs [28]. Further, the National Research Council and the Institute of Medicine reported that income, education, occupation, sex, and race/ethnicity were vital social factors that are directly connected to chronic diseases [29]. Moreover, previous studies with British men and women (civil government employees) found that those with the highest professional rank had the lowest percentage of deaths (regardless of the cause) [9, 30].

A ten-year follow-up study found that the incidence of chronic diseases increased when both men and women were overweight/obese [31]. Another study reported that US residents aged 55 to 64 years were more vulnerable to chronic diseases, particularly diabetes and heart disease than were their British counterparts, even after adjusting for age and behavioral risk factors such as for overweight/obesity, smoking, and heavy drinking [32]. This may be due to the excessive rates of obesity across the US, especially among those with less education and income less than 50,000 US dollars.

It is well-established that smoking is associated with at least 12 types of cancer, six types of cardiovascular disease, diabetes, COPD, and other chronic diseases [8]. These findings corroborate with the current results and the results of a previous report [33]. According to that report, cause-specific mortality from several diseases among a sizeable contemporary population in the US was two to three times higher among current smokers than those who never smoked. An interesting finding to note that smoking status was not associated with asthma. Evidence shows that some studies showed that the development of asthma was associated with active cigarette, but not all studies [34]. It has been concluded that the interaction between smoking and asthma remains answered by many research questions [34].

Also, the alcohol consumption rate in America is one of the highest in the world. This was associated with cancer, diabetes mellitus, and heart diseases [35]. The rates of alcohol-attributable net deaths per 100,000 people in the US are 8.8 for men and 1.6 for women [35]. Results from a population-based cohort study suggest that alcohol intake increases the risk of coronary heart disease [36].

Although all these studies have varied methodologies and populations, the present results are significant in the following major respects. This is the first study that examined the association between social determinants and chronic diseases among people at high risk for knee osteoarthritis [21]. Moreover, the present study used data from a large, multi-centered, observational study that recruited participants from four urban cities across the US. Furthermore, data from a large sample were examined using multivariable logistic regression analyses.

Nonetheless, some limitations must be reported. This study employed in specific US cities; therefore, causation cannot be inferred, and the results cannot be generalized to other regions. The findings may be strengthened if included some other factors that may influence chronic diseases such as physical activity and a sedentary lifestyle. Further, participants’ self-reported responses may have led to a recall bias that may affect the results. Chronic diseases were not specific such as diabetes (type 1 or type 2). The duration of chronic diseases, year of heart attack, and the number of times had a heart attack, are another limitation that may affect our findings and the generalizability of the results. It is essential to bear in mind that we have not analyzed the data by median personal income by educational attainment, the number of years unemployed, stayed single or with family. Overall, this study’s results must be interpreted with caution because the selection of social determinants and the way of these variables being analyzed may limit the validity of the findings.

## Conclusions

This study was aimed to examine the association between social determinants and the presence of chronic diseases. The results revealed that social determinants are associated with chronic conditions, such as arthritis, asthma, cancer, COPD, diabetes, and heart attack. These findings can inform public health strategies in the US because some of these social determinants are modifiable or treatable. Future longitudinal research is required to confirm this relationship by examining the critical factors for chronic diseases, such as glycemic control, type and degree of cancers, and duration of chronic diseases including biological and environmental factors like unhealthy ozone levels and air pollutants.

## Data Availability

The datasets generated and/or analyzed during the current study are publicly available in the National Institutes of Health repository, https://oai.nih.gov.

## Abbreviations

OAI: Osteoarthritis Initiative
COPD: Chronic obstructive pulmonary disease (COPD)
US: United States
QoL: Quality of life
SAS: Statistical analysis software
ICC: The Charlson comorbidity index
BMI: Body mass index
MRI: Magnetic resonance imaging
MD: Maryland
PA: Pennsylvania
RI: Rhode Island
OH: Ohio
